# Ethical Challenges for an Understanding of Suffering: Voluntary Stopping of Eating and Drinking and the Wish to Hasten Death in Advanced Patients

**DOI:** 10.3389/fphar.2018.00294

**Published:** 2018-03-29

**Authors:** Andrea Rodríguez-Prat, Cristina Monforte-Royo, Albert Balaguer

**Affiliations:** ^1^Faculty of Humanities, Universitat Internacional de Catalunya, Barcelona, Spain; ^2^Department of Nursing, School of Medicine and Health Sciences, Universitat Internacional de Catalunya, Barcelona, Spain; ^3^School of Medicine and Health Sciences, Universitat Internacional de Catalunya, Barcelona, Spain

**Keywords:** palliative care, advanced patients, end of life care, qualitative research methods, wish to hasten death, voluntarily stopping eating and drinking, ethics

## Abstract

Some persons with advanced disease but no significant cognitive impairments consciously decide to stop taking food and fluids orally, even though they remain physically able to do so. The question is to what extent voluntarily stopping eating and drinking (VSED) may be considered an expression of a wish to hasten death, in the sense that the latter has been defined recently. We analyze the data reported in some studies in relation to primary care patients who died as a result of VSED and examine their results in light of the qualitative findings of patients that expressed a wish to die. In our view, VSED can be understood as a response to physical/psychological/spiritual suffering, as an expression of a loss of self, a desire to live but not in this way, a way of ending suffering, and as a kind of control over one’s life. Thus, VSED is consistent with the wish to hasten death. Prior to interpreting this act as a deliberate expression of personal autonomy, it is important to explore all possible areas of suffering, including physical symptoms, psychological distress, existential suffering, and social aspects. Failure to do so will mean that we run the risk of abandoning a fellow human being to his or her suffering.

Voluntarily stopping eating and drinking (VSED) is a topic that raises challenging clinical and ethical questions. VSED defined as “an action of a competent, capacitated person, who voluntarily and deliberately chooses to stop eating and drinking with the primary intention to hasten death because unacceptable suffering persists” (Ivanović et al., 2014) leads to crucial questions about clinical ethics and clinical practice: what is the quality of life of the patients who prefer to die than to carry on living? What is the good care to provide to these patients and what is the good for them? What factors contribute to some patients wanting to die? Should this be understood as an unequivocal case of the wish to hasten death (WTHD), as a request to end their life?

In a recent study carried out by Bolt et al. (2015) in the Netherlands, the role and involvement of family physicians when confronted with patients who accelerate their death by VSED was analyzed. The data from this study (Bolt et al., 2015) suggest that VSED is a decision taken by adult or older patients with severe disease, short life expectancy, and dependency on others for everyday care. Importantly, these people are physically able to take in food and fluids orally, but they are consciously unwilling to do so.

The statements made in this article led us to reflect on whether VSED can properly be considered a particular case of the WTHD and, if it is the case, what the implication would be for clinical practice. Our reflection is based on the systematic review and synthesis of qualitative studies (meta-ethnography) on the meaning of the WTHD in patients with advanced disease carried out by our group (Monforte-Royo et al., 2012). In this systematic review we analyzed seven qualitative studies carried out in Australia, Canada, China, and United States. The characteristics of these studies are shown in **Table 1**.

**Table 1:** Characteristics of the studies included in the present review.

Source paper	Country	Participants	Main themes/results	Setting
Lavery et al., 2001	Canada	Thirty one men; one woman with HIV/AIDS	(1) Disintegration (related to loss of function)	HIV Ontario Observational Database
			(2) Loss of community	
			(3) Loss of the self	
Kelly et al., 2002	Australia	Thirty terminally ill cancer patients	(1) Physical symptoms	Inpatient hospice unit and home PC^∗^ service
			(2) Psychological suffering	
			(3) Burden to others	
			(4) Demoralization	
			(5) No satisfaction with life experiences	
Coyle and Sculco, 2004	United States	Seven terminally ill cancer patients	The WTHD as:	Pain and PC^∗^ unit in an urban cancer research center
			(1) A manifestation of the will to live	
			(2) A dying process so difficult that an early death was preferred	
			(3) An intolerable immediate situation	
			(4) A hastened death could extract a patient from an unendurable and specific situation	
			(5) A manifestation of the last control the dying can exert	
			(6) A way of drawing attention to “me as a unique individual”	
			(7) A gesture of altruism	
			(8) An attempt at manipulation of the family	
			(9) A despairing cry	
Mak and Elwyn, 2005	China	Six patients	(1) Reality of the disease progression	Twenty six-bed hospice
			(2) Perception of suffering for self and significant others	
			(3) Anticipation of a future worse than death itself	
			(4) Desire for good quality end-of-life care	
			(5) Holding environment	
Pearlman et al., 2005	United States	Thirty five patients	(1) Illness-related experiences	Patient advocacy organizations that counsel persons interested in AS, hospices and grief counselors
			(2) Loss of their sense of self	
			(3) Fears about the future	
Schroepfer, 2006	United States	Eighteen terminally ill elders	Frames toward dying:	Two PC programs, two hospital outpatient clinics, and six hospices
			(1) Neither ready nor accepting	
			(2) Not ready but accepting	
			(3) Ready and accepting	
			(4) Ready, accepting, and wishing death would come	
			(5) Considering a hastened death but having no specific plan	
			(6)Considering a hastened death with a specific plan	
Nissim et al., 2009	Canada	Twenty seven ambulatory cancer patients	(1) WTHD as a hypothetical exit plan	Outpatient clinics at a large cancer center
			(2) WTHD as an expression of despair	
			(3) WTHD as a manifestation of letting go	

*^∗^PC, palliative care*.

For the present analysis we also have taken into account a recent consensus definition of the WTHD in which we participated. This proposal understands that “the WTHD is a reaction to suffering, in the context of a life-threatening condition, from which the patient can see no way out other than to accelerate his or her death” (Balaguer et al., 2016). Our goal is to contribute to the understanding of patients who voluntarily stop eating and drinking, and, therefore, to help improve the practice of physicians who are responsible for their care. In what follows, we will also try to compare the data regarding the VSED and the results derived from our meta-ethnography which highlighted that any expression of the WTHD was underpinned by suffering (Monforte-Royo et al., 2012). This suffering was seen as a response to physical-psychological-spiritual-existential impairment. Poor-quality dying experiences –related to an inadequate management of pain and psychological and existential suffering– have contributed to people defending practices in favor of voluntary death (Schroepfer, 2006). Conversely, knowing which factors trigger the emergence of the desire to die and addressing them clinically can improve the quality of care provided for patients in palliative care. In the study of Bolt et al. (2015), different levels of suffering are likewise apparent among the patients who voluntary chose to die by VSED. The most common patient motives for hastening death reported by family physicians were somatic (79%), existential (77%), and dependence-related (58%). Other reasons mentioned included loss of sense of dignity and loss of self, social factors, and psychiatric suffering. These variables are clearly consistent with the idea of the WTHD as a reaction to physical, psychological, and/or spiritual suffering.

One conclusion to be drawn from this Dutch study (Bolt et al., 2015) is that patients with severe disease may regard VSED as a way of ending their suffering and of exerting some control over their life. Similarly, the patients considered by our meta-ethnography did not regard death as an end in itself but, rather, as a way of escaping from overwhelming suffering.

Furthermore, some patients experienced both a wish to die and a wish to go on living. This paradox is also observed among patients who chose to die by VSED (Bolt et al., 2015), since the dying phase is then both prolonged and potentially reversible. This reversibility would appear to reflect two aspects that emerged in our study (Monforte-Royo et al., 2012), namely the possibility of controlling when and how one dies (“to have an ace up one’s sleeve just in case”) and the “desire to live but not in this way.” Again, we see the idea that death was not always what the patients wished for but it can also mean to end a life that apart from the context of their illness would have been wanted.

Some of the points made by Quill (2015) regarding VSED are also consistent with the findings of our meta-ethnography (Monforte-Royo et al., 2012). For example, he mentions the idea of assisted suicide (AS) as a “last resort” when faced with extreme suffering. The proximity of inevitable death can lead some persons to experience an intense need for control, and in such cases, taking decisions about how one lives or dies may become a good control strategy that reduces emotional distress or even fosters an improved sense of well-being. In this context, the more extreme the suffering the greater may be the need for control, and thus it becomes more likely that a person will take desperate decisions (i.e., as a last resort). For a person in such a situation, being able to decide for oneself how and when one dies may be experienced as a form of self-determination, as preserving what little is left of the life he/she once had.

A similar paradox is present in other observation made by Quill (2015), since he suggests that for patients like the one he describes AS may have more to do with self-preservation than self-destruction. However, it is worth remembering that a common experience reported in studies of advanced patients who request AS (Lavery et al., 2001; Chochinov et al., 2002; Pearlman et al., 2005) is a “loss of the self,” the sense that one’s own essence or identity is disintegrating (“I’m no longer the person I was before the illness”). Within this framework, therefore, the WTHD would correspond to a manifestation of the desire to flee from a reality dominated by suffering, and thus it is perhaps accepts the idea of destructing a disintegrating self rather than the wish to assert one’s own individuality. In many cases, as stated above, the idea of a loss of self is associated with a perceived loss of sense of dignity and meaning in life. This raises the question as to what extent the decision — based on personal autonomy — to end one’s life can be regarded as an act of self-preservation.

Having compared the findings reported in the Dutch study (Bolt et al., 2015) with those of the meta-ethnography (Monforte-Royo et al., 2012) and the consensus definition of the WTHD (Balaguer et al., 2016) we confirm that VSED can be regarded as a particular expression of the WTHD. Hence, like the WTHD, the act of VSED should be understood as being underpinned by one or more kinds of suffering, the causes of which would need, in clinical practice, to be identified and, if possible, addressed in some way. Although, in the Dutch study (Bolt et al., 2015) only 13% of the 99 patients who died by VSED were said to have reported depressive symptoms, studies that have analyzed predictors of the WTHD in advanced patients have found that depression is one of the strongest predictors (Chochinov et al., 1995; Villavicencio-Chávez et al., 2014). Due to the retrospective nature of the study (Bolt et al., 2015), however, it is not known whether any attempts were made by family physicians to rule out this factor or, in the event that it was present, to provide treatment. The evidence to date (Breitbart et al., 2000; Villavicencio-Chávez et al., 2014) suggests that proactive intervention in relation to the WTHD may be a crucial step in alleviating the suffering experienced by many patients and in widening the focus beyond the desire to die, which has also been described as a “cry for help” (Coyle and Sculco, 2004; Nissim et al., 2009; Monforte-Royo et al., 2012).

From an ethical perspective, the physicians should not hasten the death of a patient. When faced with a patient who refuses food or fluids but does want to receive medical treatment to relieve the dying process that comes as a consequence, the hospital has the moral and professional authority to not accept this decision. Seeking out death as an end is different from the patient dying as an indirect results of a medical intervention (the theory of the double effect).

Recent trends suggest that the VSED can be described as a treatment option rather than as an activity undertaken by a patient on his/her own (Jansen, 2015; Quill et al., 2018). Viewed in this way, namely as an option that can be potentially supported by the clinician, its ethical consideration would be very similar to that of AS. In this context, supporters of VSED have proposed that the practice be brought under the umbrella of standard care, whereas those who oppose it fear that this would lead physicians to regard it as just another viable option for those patients who, due to their suffering, wish to put an end to their life. Therefore, in terms of the ethical challenge posed by VSED, there is a need, as with the WTHD, to do all we can to understand what lies behind this complex phenomenon. For if ethics implies an inquiry into what we are capable of doing or not doing, and into how the greater good may be achieved, then we need to explore further what it means when someone expresses the wish to die.

In instances of patients with VSED due to poor symptom control, it will usually be possible to use other measures to relieve their suffering: a saline drip for hydration or other comfort measures. When faced with a patient who has taken this decision (reflexive), as a free choice, we could consider that this choice would no longer involve the medical science. Where the patient presented a depressive disorder, it would be necessary to treat the depression first as this may be what is behind their decision.

The analysis of the qualitative studies about this phenomenon allow us to understand that when a patient expresses one way or another that they wish to die, it is necessary to look at the reasons and meaning behind this desire in more depth. The retrospective studies offer valuable information in terms of determining the clinical precursors of any phenomenon. However, it is necessary to look more closely at the experience of these patients and to diagnose psychiatric disorders, that so frequently occur in this stage, proactively and as early as possible.

In every case it should be determined whether the WTHD is either part of a clinical mental condition (depression or psychological impairments), an expression of wish to end a process of disintegration, etc. Even if such conditions are ruled out, it would be wise, prior to interpreting this act as a deliberate expression of personal autonomy as the ‘Right to Die’ movements suggests, to explore all possible areas of suffering, including physical symptoms (either present or foreseen), psychological distress, existential suffering, and social aspects. Otherwise, respecting the autonomous desire of the patient could be at the detriment of providing good care for and determining the good of the patient in all those cases in which there is a treatable condition. Failure to do so would mean that we run the serious risk, both as health professionals and as human individuals, of abandoning a fellow human being to a fate in which suffering pervades.

## Author Contributions

AR-P and CM-R designed the study. AR-P, CM-R, and AB wrote the manuscript, made substantial contributions to the identification of relevant literature, the interpretation of findings and were involved in drafting the manuscript and revising it critically. All authors gave final approval to this manuscript.

## Conflict of Interest Statement

The authors declare that the research was conducted in the absence of any commercial or financial relationships that could be construed as a potential conflict of interest.
